# CMOS-Integrated Film Bulk Acoustic Resonators for Label-Free Biosensing

**DOI:** 10.3390/s100504180

**Published:** 2010-04-27

**Authors:** Martin Nirschl, Arto Rantala, Kari Tukkiniemi, Sanna Auer, Ann-Charlotte Hellgren, Dana Pitzer, Matthias Schreiter, Inger Vikholm-Lundin

**Affiliations:** 1 Siemens AG Munich, Corporate Technology, Otto-Hahn-Ring 6, 81739 Munich, Germany; E-Mails: Dana.Pitzer@siemens.com (D.P.); Matthias.Schreiter@siemens.com (M.S.); 2 Laboratory of Biosensors and Bioelectronics, Institute for Biomedical Engineering, ETH Zurich, Switzerland; 3 VTT Technical Research Centre of Finland, Tietotie 3, Espoo, FI-02044 VTT, Finland; E-Mails: Arto.Rantala@vtt.fi (A.R.); Kari.Tukkiniemi@vtt.fi (K.T.); 4 VTT Technical Research Centre of Finland, P.O. Box 1300, FI-33101 Tampere, Finland; E-Mails: Sanna.Auer@vtt.fi (S.A.); Inger.Vikholm-Lundin@vtt.fi (I.V.-L.); 5 Biosensor Applications AB, Solna Strandväg 3, 171 54 Solna, Sweden; E-Mail: Ann-Charlotte.Hellgren@biosensor.se

**Keywords:** FBAR, film bulk acoustic resonator, CMOS, DNA, thickness shear mode, TSM, label-free, biosensor, biomolecular interaction analysis

## Abstract

The throughput is an important parameter for label-free biosensors. Acoustic resonators like the quartz crystal microbalance have a low throughput because the number of sensors which can be used at the same time is limited. Here we present an array of 64 CMOS-integrated film bulk acoustic resonators. We compare the performance with surface plasmon resonance and the quartz crystal microbalance and demonstrate the performance of the sensor for multiplexed detection of DNA.

## 1. Introduction

Biomolecular interaction analysis is an important method for drug discovery and drug development because it allows quantification of the interaction of a wide range of biomolecules with high sensitivity and low sample consumption [1]. A label-free sensor is required to study the interaction between molecules because the presence of a label might alter the interaction process.

A wide variety of optical and acoustic label-free biosensors is known: Surface Plasmon Resonance (SPR) is the most widely used technology [2], but there are available or emerging alternatives: Optical Waveguide Light-mode Spectroscopy (OWLS), Bio-Layer Interferometry (BLI), ellipsometry, thickness shear mode resonators like the Quartz Crystal Microbalance (QCM) and Film Bulk Acoustic Resonators (FBAR), Surface Acoustic Wave (SAW) devices and microcantilever based devices.

Each has certain advantages and disadvantages: SPR has a very high sensitivity [3], but a metal is needed for plasmons to occur. Gold is mostly used in SPR, but QCM also has a gold electrode. With both techniques, surface interactions can in principal be followed on all kinds of surfaces if the gold surface is coated with a thin layer. On QCM, also thicker layers e.g. several hundreds of nanometers of an oxide can be used, which is why QCM is commonly used as a thickness monitor in thin-film deposition processes. However, QCM operated in liquid measures not only the adsorbed mass but also the liquid coupled to the adsorbent, which might make a quantitative sensing difficult in liquid phase measurements [4]. OWLS allows determination of the adsorbed mass and the refractive index of the adsorbent at the same time [5] but it requires a highly transparent surface [6]. BLI requires very low sample volumes because it does not need a flow cell [7]. As a disadvantage, the costs of the consumables are high [8]. Ellipsometry allows highly sensitive measurements but the necessary equipment is very expensive [9]. SAW resonators have the highest theoretical mass sensitivity among the acoustic resonators because the energy is maximized near the surface [10]. This, on the other hand, makes it difficult to use SAWs in liquids because a strong damping occurs. The microcantilever is a simple device, which can be produced in large arrays at low cost. The difficulties in reading out the transducer with low noise and minimized influence of artifacts might be mentioned as a main disadvantages [11].

FBARs operating in shear mode have been produced previously for application in a liquid environment [12–14]. These resonators were operating in shear mode rather than in the longitudinal mode, which significantly increases the quality factor of the resonator when used in liquid environment. In order to excite acoustic shear waves, c-axis inclined piezoelectric thin-films made of zinc oxide and aluminum nitride have been developed and studied intensively [15–22].

FBARs can be produced at low cost using standard thin-film technology. They are binding sensitive which means that substances bound to the surface cause a shift in the resonance frequency. Thus the surface layer has to be very specific, as also substances non-specifically bound to the surface will be detected. Optical sensors detect changes in refractive index in the proximity of the sensor surface and a reference surface is often used to discriminate non-specific binding. Also acoustic sensors need a reference surface if unspecific binding significantly contributes to measured signal. The number of pixels that can be used is however large.

We have previously presented FBARs as a label-free biosensor with promising properties such as a sensitivity similar to that of QCM [23]. However, those FBARs made use of a network analyzer for read-out of the electronic signal. This is costly and makes it difficult to access many pixels at the same time. In order to obtain the response from a large number of pixels, a small and cheap read-out circuit specifically designed for the purpose of determining the resonance frequency of a multiplicity of resonators is necessary. Interface circuits for the QCM for application in air and liquid have been developed and optimized for decades [24]. The development of a read-out circuit for FBARs, however, has different prerequisites than for the QCM because of the generally lower quality factors of the FBAR compared to the QCM and the higher variation of the resonance frequencies among different resonators originating from the FBAR production process. While oscillator based read-outs were previously presented [25,26], an impedance-based read-out is more robust especially in liquid environment. Schneider *et al*. [27] presented a design utilizing a direct digital synthesis, where a test signal is generated using a digital signal processor, D/A-converted and the corresponding power is measured. As an additional advantage of this method, not only the resonance frequency but also the changes in the energy dissipation can be determined. This can be useful to determine viscoelastic properties of adsorbents [28,29].

This direct digital synthesis leads to a highly complex readout circuitry and the maximum frequency band is quite limited. The importance of a simple readout is emphasized when the impedance analysis is utilized for an integrated sensor matrix. A novel, simple, but accurate impedance change detection method was developed for FBAR resonators. The core part of the analyzer is a ring oscillator based voltage-controlled oscillator (VCO). The VCO frequency is swept over a large frequency band and the FBAR impedance response is acquired from the VCO output. A dedicated algorithm resolves the series and parallel resonance frequencies of the FBAR.

In this paper, we present arrays with 64 acoustic resonators on one chip, which are monolithically integrated into a novel impedance-based CMOS. The sensitivity of the device is determined and the usability for label-free DNA detection is demonstrated.

## 2. Experimental Section

### 2.1. CMOS-Integrated FBAR Array

The FBARs consist of a piezoelectric ZnO thin-film with the c-axis inclined from the layer normal. The ZnO deposition process used for the FBARs used in this study is described in detail in [30]. The ZnO layer was sandwiched between two electrodes and mounted on top of an acoustic mirror in order to acoustically separate the resonator from the substrate. Acoustic vibrations were excited when an alternating electric field was applied to the electrodes. The fundamental resonance frequency of the FBAR was around 800 MHz and decreased when additional mass was added on top of the resonator. The FBARs were back-end processed in arrays of 4 × 16 pixels on active 0.35 μm CMOS wafers. Under each FBAR pixel there was a dedicated read-out circuit. This circuit included an interface block which provided communication with the system level, a voltage-controlled oscillator (VCO), a local control which interprets the system level commands and controls the VCO, and a frequency meter (digital counter). The resonance frequency acquisition is performed as follows: A value for the VCO control is calculated at system level and applied to the corresponding pixel. The corresponding frequency is obtained by integrating the VCO output with the digital counter.

At the beginning of the analysis the operation point for each FBAR pixel had to be set. For this, the control for the VCOs was swept along a range around the resonance frequency while the output frequency in recorded. The acquired control voltage versus frequency curve carries information about the electrical impedance seen by VCO (*i.e.*, the FBAR sensor) [31]. An empirical algorithm was developed to detect the operation point corresponding to the parallel resonance frequency of the resonator.

Once the operation point for all pixels has been acquired, the control value for VCO was fixed during the measurement. During the measurement the output frequencies of the VCOs were continuously measured and the observed frequency deviations reflect the changes of the FBAR resonance frequency.

**Figure 1.**
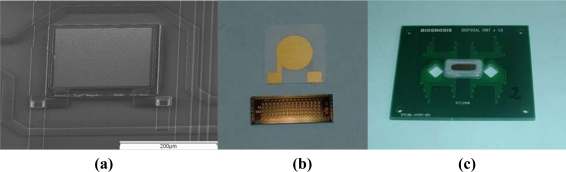
(a) A scanning electron microscope picture of one of the FBAR pixels. The pixel size is 200 μm × 200 μm. (b) FBAR chip under a QCM crystal. While the physical working principle is similar, the FBAR allows integrating 64 pixel on about the same area like a QCM crystal. (c) The CMOS-integrated FBAR packaged on a credit-sized board, which allows a very simple handling of the sensor.

The finished chips were then glued onto a printed circuit board, wire bonded and sealed. About a dozen of chips with all 64 pixels working were available from one 6” wafer. A SEM picture of one of the resonators can be seen in Figure 1a. Figure 1b shows the complete chip with 64 pixels under a quartz crystal. The cartridge (Figure 1c) contained a flow cell (about 10 μl volume) with a simple inlet and outlet, which allowed manual injections of the liquids using a syringe. A minimum amount of 500 μl was injected to ensure complete exchange of the liquids.

### 2.2. Chemicals

Tris(hydroxymethyl)aminomethane, 6-mercapto-1-hexanol (MCH), bovine serum albumin (BSA), minimum 98% purity, were purchased from Sigma Aldrich Finland Oy (Helsinki, Finland). *N,N*-bis (2-hydroxyethyl)-α-lipoamide (Lipa-DEA) was prepared as previously described [32]. The reagents EDTA and Na_2_HPO_4_ were purchased from Merck KGaA; sodium chloride and NaH_2_PO_4_ from J. T. Baker.

Probes disulfide-modified in the 5′ end by a dimethoxytrityl-group (5′-DMT-S-S-(CH_2_)_6_-DNA) with sequences of 5′-CGA TTG TAT TCG GAT AGG ATT TTA TGG and 5′-GCT TCC GAT CAC ACT CAT TTA CAC were used. The probes are referred to as S-S-PTGS2 and S-S-CALCA. Single-stranded (ss), complementary DNA molecules with sequences of 5′-CCA TAA AAT CCT ATC CGA ATA CAA TCG (PTGS-27) and 5′-TGT GTA AAT GAG TGT GAT CGG AAG C (CALCA-25) were used. PCR amplified products had a length of 92 and 123 base pairs for CALCA and PTGS2, respectively. The probes were selected due to their relevance in breast cancer diagnosis [33]. All oligos were custom synthesized from Metabion (Martinsried, Germany). A phosphate-buffered saline (PBS) buffer solution of 20 mM Na_2_HPO_4_/NaH_2_PO_4_, 300 mM NaCl, 1 mM EDTA, pH 7.5 was used in all measurements.

### 2.3. BSA Measurements

A 10 MHz QCM Biosens system (Biosensor Applications AB, Sweden) read-out at the fundamental frequency and a Biacore 3,000 instrument (Biacore AB, Uppsala, Sweden) were used for physisorption studies with BSA. Glass slides coated with a 50 nm thin film of gold by sputter coating (using an Edwards E306A sputter coater) were cleaned in a hot hydrogen peroxide/ammonium hydroxide/water solution (1/1/5) and rinsed with water. The slides were mounted in a plastic chip cassette by double-sided tape and inserted into the Biacore instrument.

BSA at concentrations ranging from 1–1,000 μg/mL was applied on the gold surfaces of the devices while recording the change in refractive index and resonance frequency for SPR, QCM and FBAR, respectively. The lowest BSA concentration was added after a stable baseline had been recorded for 1 minute for SPR and at least 5 minutes for QCM and the FBAR. Then alternately buffer and BSA in increasing concentrations were injected for 5 minutes each. On SPR, the flow speed was 20 μL/min, on QCM and FBAR, the liquids were injected using a syringe and without flow. On all sensor systems, both buffer and BSA stayed in contact with the sensor for 5 minutes each. The measurement was performed at 25 °C on SPR and the passive FBAR. Room temperature was used for the CMOS-integrated FBAR and on the QCM.

### 2.4. Functionalisation of the Gold Surfaces

Binary solutions of the probes, the S-S-CALCA and S-S-PTGS2 at a concentration of 7 μM and Lipa-DEA at a concentration of 700 μM, were dispensed on the FBAR gold surfaces. The two functionalisations are referred to as S-S-CALCA/Lipa-DEA and S-S-PTGS2/Lipa-DEA. Reference pixels were spotted only with 700 μM Lipa-DEA. A piezo dispenser (sciFLEXARRAYER S5, Scienion AG, Germany) was used for the FBAR spotting process. The spotting procedure was performed at 15 °C and 50% humidity. The functionalized chips were kept in the same environment for at least one hour after the spotting in order to give a sufficient time for the probes to bind to the gold surface. The FBARs were rinsed with water, dried and stored for 1 to 3 days at +4 °C.

About 1 nL drop volume was sufficient to cover the complete squared gold electrode and not to leave parts of the gold surface uncovered which would significantly increase unspecific binding. The drops also remained separated on the chips so that the different localized functionalisations did not intermix on the chip (Figure 3a).

FBAR measurements were started by recording a baseline for at least 5 minutes followed by injection of the sample with an interaction time of 15 minutes, if not stated otherwise. The surface was rinsed with buffer for 10 minutes. If more than one concentration of the complimentary DNA was measured, the surface was regenerated by rinsing the surface twice for 2 minutes each with 10 mM NaOH-0.1% SDS-solution with a 2 minutes buffer rinse in between. Human blood serum (Sigma Aldrich) was diluted 1:100 with buffer in order to avoid measurement artifacts caused by changes in viscosity or refractive index.

## 3. Results and Discussion

### 3.1. Mass Sensitivity Comparison Obtained with FBAR, SPR and QCM

**Figure 2.**
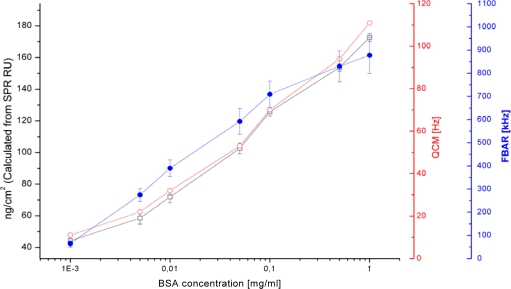
Titration curves for bovine serum albumin as measured with QCM ( 
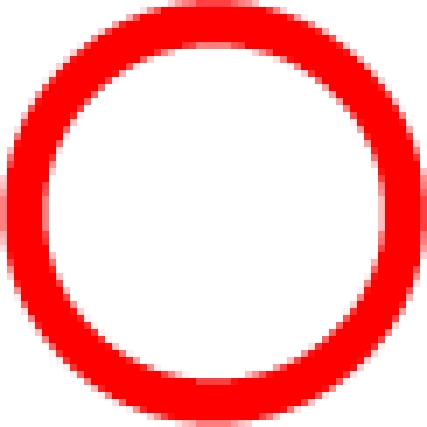
), CMOS-integrated FBAR ( 
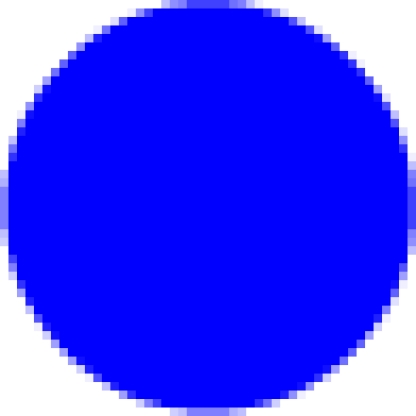
) and SPR (□). The curve for the FBAR measurement is the average of 10 pixels, the SPR represents 4 channels; the error bars show the standard deviation.

In order to experimentally determine the mass sensitivity of the FBAR, a reference measurement was conducted with the FBAR, QCM and SPR. For this, all sensors were exposed to BSA in concentrations ranging from 1–1,000 μg/mL. All three sensors show the same trend, *i.e.*, an increase in response with increased concentrations. Figure 2 shows the resulting titration curve of the CMOS-integrated FBAR, QCM and SPR.

The SPR results were used to determine the adsorbed surface mass obtained for each BSA concentration because the response in resonance units can be easily converted into surface mass: One resonance unit corresponds to a surface mass of 0.1 ng cm^−2^ [34]. With the amount of BSA adsorbed to the surface known for each concentration, from the corresponding frequency shifts of FBAR, the mass sensitivity in terms of frequency shift per adsorbed mass was calculated. The same calculation was done for the QCM in order validate the results as the mass sensitivity for the QCM, contrary to the FBAR, can be calculated using the Sauerbrey equation. Table 1 summarized the mass sensitivity, the noise of the measurements and the resulting limit of detection. The experimentally obtained mass sensitivity of the QCM is about three times higher than the value obtained using the Sauerbrey equation. This result is as expected because the QCM unlike the SPR does not only sense the adsorbed protein but also the liquid coupled to it [35]. This suggests that this experimental method is capable to determine the mass sensitivity of the FBAR. The mass sensitivity of the FBAR is nearly two orders of magnitude higher than the sensitivity of the QCM.

This is in agreement with what has been shown previously [13]. However, in order to properly evaluate the FBAR sensor performance, it has to be taken into account that also the noise is higher by nearly two orders of magnitude [36]. The resulting limit of detection (LOD) in terms of the smallest surface mass that can be detected is defined as the frequency shift per surface mass divided by three times the frequency noise over 10 measurements. The resulting LOD is similar for QCM, the passive and CMOS-integrated FBARs. The SPR has a 7–25 fold lower LOD than the acoustic resonators investigated.

The standard deviation between several FBARs is higher than the noise by the resonators and also higher than the standard deviation of the measurements obtained with SPR. Also, the curves recorded with QCM agree much better to the one from SPR while the curve from FBAR shows a slightly different behaviour. As there were air bubbles visible in the flow cell used with the FBAR and some resonators showed frequency jumps during the injection of liquids, the main influence for the differences in the signal and the higher standard deviation might be air bubbles in the vicinity of the resonator surface. The results are therefore quite promising taking into account that Biacore 3,000 uses a highly developed fluidic system whereas no emphasis was put on the FBAR fluidics and the FBAR could moreover be further improved.

### 3.2. Multiplexed DNA Measurement

**Figure 3.**
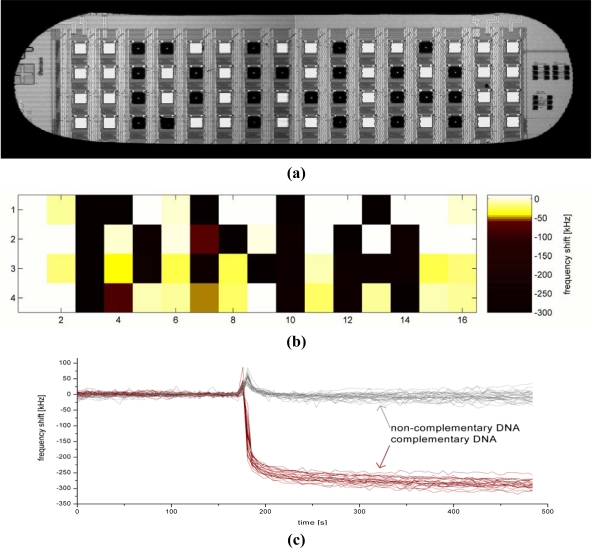
(a) The FBAR array with some of the pixels functionalized. The black squares are drops of liquid containing probes and Lipa-DEA. The squares appearing in white are uncovered gold pixels. (b) Frequency shift of all 64 pixels after the addition of the complimentary DNA at saturation. (c) The frequency curves of 50 selected pixels. The complementary DNA was added at t = 180 s.

In order to demonstrate the high number of pixels that can be used at the same time, a pattern was spotted on the sensor array. Figure 3(a) shows the 4 × 16 pixel array after the S-S-CALCA/Lipa-DEA solution was spotted on some pixels, which appear in black. The pixels appearing in white are the blank gold surfaces and were coated in a subsequent step with S-S-PTGS2/Lipa-DEA that was used as a reference coating. The arrangement of the pixels represents the letters DNA.

Figure 3(b) shows the sensor response of all 64 pixels at saturation after hybridisation with the complementary DNA (CALCA). A frequency shift of −270 ± 80 kHz was observed for the pixels functionalized with the probes after a 5 minutes interaction time. No frequency shift (2 ± 17 kHz) could be observed for the pixels passivated with Lipa-DEA. Figure 3(c) shows the time resolved frequency shifts of 50 pixels. The remaining 14 pixels on the chips are not shown because their resonance frequency showed high positive frequency shifts upon the injection. Together with the injection, like in the previous measurement with BSA, air bubbles could be seen in the flow cell, which are likely to be the reason for the failing resonators.

### 3.3. Multiplexed Measurement of PCR Amplified Products in Buffer and Serum

**Figure 4.**
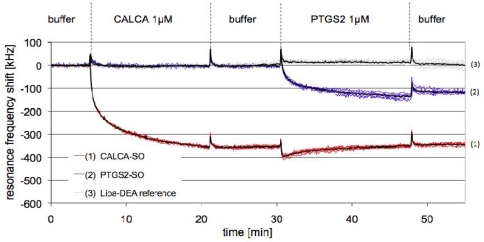
The frequency response of selected pixels of one FBAR chip: The red curves correspond to surfaces functionalized with S-S-CALCA/Lipa-DEA, the blue curves to surfaces functionalized with S-S-PTGS2/Lipa-DEA and the grey curves to surfaces passivated with Lipa-DEA only. The number of replicas is 5 for each type of functionalisation; the black curves are the average of the 5 curves.

Resonator arrays with three different functionalisations were prepared: S-S-PTGS2/Lipa-DEA, S-S-CALCA/Lipa-DEA and Lipa-DEA only. Figure 4 shows the different frequency responses obtained for pixels functionalized with S-S-CALCA, S-S-PTGS2 and blocked with Lipa-DEA on interaction with complementary DNA.

When the complimentary CALCA PCR product at a concentration of 1 μM was added (t = 5 min) a change in resonance frequency corresponding to −332 ± 3 kHz was observed. The response was obtained only for the resonators functionalized with S-S-CALCA/Lipa-DEA, while the frequency shifts for the pixels coated with only Lipa-DEA or S-S-PTGS2/Lipa-DEA were below noise. A frequency shift of −130 ± 10 kHz was observed for the S-S-PTGS2/Lipa-DEA functionalized pixels after adding a 1 μM solution of the PTGS2 PCR product (t = 30 min). The hybridization was previously found to be lower for PTGS2 than for CALCA [33]. Again, no frequency shift was observed for the Lipa-DEA passivated pixels and for the pixels functionalized with the non-complimentary probes.

**Figure 5.**
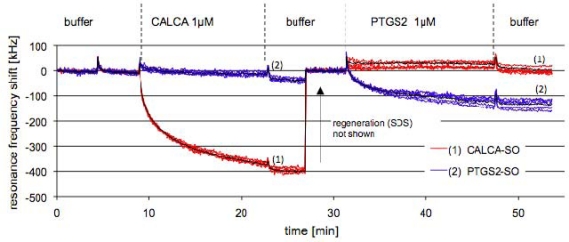
The frequency response of selected pixels of one FBAR chip: The red curves (5 pixels) show responses for surfaces functionalized with S-S-CALCA/Lipa-DEA and the blue curves (5 pixels) for surfaces functionalized with S-S-PTGS2/Lipa-DEA. The black curves represent the average of the 5 curves.

For the measurement in serum, a chip was functionalized with S-S-CALCA/Lipa-DEA and S-S-PTGS2/Lipa-DEA. Figure 5 shows the FBAR response on the addition of diluted serum spiked with the complementary PCR products. A high frequency shift of −360 ± 20 kHz was observed when pixels functionalized with the complementary sequence were allowed to interact with a serum solution spiked with a CALCA PCR product to a concentration of 1 μM (1:100). The frequency shift of the pixels functionalized with the non-complementary probes was below noise. The surface was then regenerated. This data was removed from the measurement curve for clarity because the viscosity changes caused high frequency jumps. The resonance frequency returned to the value of the buffer baseline showing that the hybridized DNA strand had been effectively removed. On addition of the PTGS2 PCR product at a concentration of 1 μM, a frequency shift of −130 ± 20 kHz was obtained, which was smaller than the one for the CALCA. This is in agreement with the measurement in buffer. Instead no hybridization occurred on the pixels functionalized with S-S-PTGS2/Lipa-DEA showing that there was no binding of non-complementary strands.

**Figure 6.**
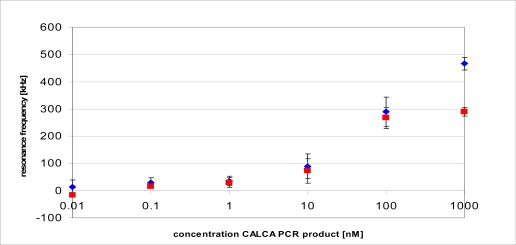
Titration curve for hybridisation of a CALCA PCR product in buffer ( 
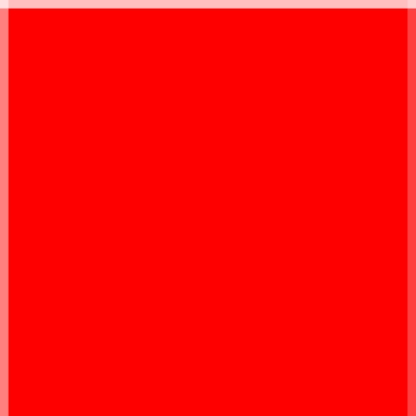
) and serum ( 
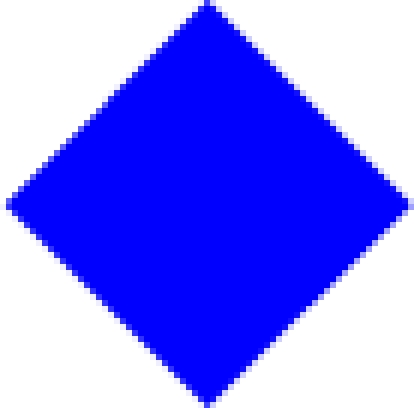
) (1:100) with a surface layer of S-S-CALCA/Lipa-DEA. The error bars show the standard deviation over 4 pixels. All points were recorded on the same chip.

Additionally, a titration curve was recorded for the S-S-CALCA/Lipa-DEA pixels in buffer and serum (1:100) for the complimentary PCR product from 10 pM-1 μM. The surface was regenerated by washing the layer with a SDS-NaOH solution between the different concentrations. The frequency shifts at saturation are shown in Figure 6 for buffer and serum for four selected pixels. The error bars show the standard deviation over the frequency shift of four pixels. The titration curve is similar for the measurement in buffer and in diluted serum apart from the highest concentration (1 μM). Layers of hybridized DNA are known to form solvent rich layers [38]. Therefore differences in the viscoelastic properties of a layer containing high amounts of serum or buffer might cause the difference in the signal at 1 μM. However, it requires future investigations to find out if this effect is a problem for quantitative measurements.

There is no visible unspecific adsorption at the smaller concentrations (*i.e.*, 1 nM and lower). This suggests that the functionalisations successfully suppress the binding of serum.

## 4. Conclusions

We have presented arrays of acoustic thin-film resonators integrated in an innovative CMOS read-out circuit. Due to the simple and efficient read-out it is possible to integrate multiple acoustic resonators on one chip using standard CMOS technology and thin-film processing. The chip presented in this study had 64 resonators on an area smaller than 1 cm^2^. The sensor array showed a limit of mass detection equal to the non-integrated FBAR and the QCM. At the current state of development, SPR had a 10-fold lower detection limit. However, the FBAR has advantages compared to the optical technologies: The read-out set-up is cheaper as it does not require any optical equipment. The smaller size results in a lower liquid consumption and a higher throughput by allowing highly multiplexed measurements. In a measurement for DNA detection in buffer, the sensor worked robustly in a liquid environment. The detection of DNA in diluted serum was also success, showing that with the FBAR it is possible to conduct measurements in crude samples. The functionalisation was capable to detect two different DNA sequences without considerable unspecific binding from the human blood serum (1:100).

## Figures and Tables

**Table 1.** Mass sensitivity, frequency noise and limit of detection for SPR, QCM, the passive and the CMOS-integrated FBAR. The mass sensitivity for the acoustic resonators is relative to their resonance frequency. For the CMOS- integrated FBAR, the noise and LOD from the best pixel and the average over a 64-pixel array are shown.

	**SPR**	**QCM**	**Passive FBAR**	**CMOS-integrated FBAR (best/average)**
Mass sensitivity	10 RU cm^2^/ng [34]	61.1 ppb cm^2^/ng (measured) 22.7 ppb cm^2^/ng (Sauerbrey [37])	5.63 ppm cm^2^/ng	7.13 ppm cm^2^/ng
Noise level (3σ)	0.63 RU	23 ppb	2.3 ppm	3.0/10.8 ppm
Mass resolution (LOD)	0.06 ng/cm^2^	0.38 ng/cm^2^	0.41 ng/cm^2^	0.42/1.5 ng/cm^2^
